# Carboxypeptidase A6 in Zebrafish Development and Implications for VIth Cranial Nerve Pathfinding

**DOI:** 10.1371/journal.pone.0012967

**Published:** 2010-09-24

**Authors:** Peter J. Lyons, Leung-hang Ma, Robert Baker, Lloyd D. Fricker

**Affiliations:** 1 Department of Molecular Pharmacology, Albert Einstein College of Medicine, Bronx, New York, United States of America; 2 Department of Physiology and Neuroscience, New York University Medical School, New York, New York, United States of America; The University of Hong Kong, China

## Abstract

Carboxypeptidase A6 (CPA6) is an extracellular protease that cleaves carboxy-terminal hydrophobic amino acids and has been implicated in the defective innervation of the lateral rectus muscle by the VIth cranial nerve in Duane syndrome. In order to investigate the role of CPA6 in development, in particular its potential role in axon guidance, the zebrafish ortholog was identified and cloned. Zebrafish CPA6 was secreted and interacted with the extracellular matrix where it had a neutral pH optimum and specificity for C-terminal hydrophobic amino acids. Transient mRNA expression was found in newly formed somites, pectoral fin buds, the stomodeum and a conspicuous condensation posterior to the eye. Markers showed this tissue was not myogenic in nature. Rather, the CPA6 localization overlapped with a chondrogenic site which subsequently forms the walls of a myodome surrounding the lateral rectus muscle. No other zebrafish CPA gene exhibited a similar expression profile. Morpholino-mediated knockdown of CPA6 combined with retrograde labeling and horizontal eye movement analyses demonstrated that deficiency of CPA6 alone did not affect either VIth nerve development or function in the zebrafish. We suggest that mutations in other genes and/or enhancer elements, together with defective CPA6 expression, may be required for altered VIth nerve pathfinding. If mutations in CPA6 contribute to Duane syndrome, our results also suggest that Duane syndrome can be a chondrogenic rather than a myogenic or neurogenic developmental disorder.

## Introduction

Carboxypeptidase A6 (CPA6) is a member of the M14 family of carboxypeptidases (CPs) that cleave C-terminal amino acids from peptides and proteins [1], [2]. These enzymes are involved in a wide variety of biological processes, from food digestion to neuropeptide maturation and modulation of extracellular signaling factors [3], [4]. CPA6 is a member of the A/B subfamily of CPs, members of which are named based on their substrate specificity, with CPA-like enzymes cleaving aliphatic/aromatic amino acids, CPB-like enzymes cleaving basic amino acids, and CPO predicted to cleave acidic amino acids [2], [5]. While the physiological substrates of CPA6 are not known, human CPA6 is secreted and interacts with the extracellular matrix where it cleaves a variety of substrates including proteins, peptides and small synthetic substrates [6].

In contrast to other members of the CPA/B subfamily [3], which include pancreatic enzymes (CPA1, CPA2, CPB1), a circulating regulator of fibrinolysis (CPB2/TAFI), and a mediator of mast cell function (CPA3/mast cell CP), CPA6 has been suggested to play a role in neuronal development through its expression in the mouse olfactory bulb, cerebellum and dorsal root ganglia [1]. The expression of CPA6 posterior to the eye, suggested to be the lateral rectus muscle [1], has been of particular interest since a disruption of the human CPA6 gene was implicated in Duane syndrome [7].

Duane syndrome is a neurodevelopmental disorder in which the lateral rectus muscle, responsible for abduction of the eye, is not innervated by the abducens nerve (cranial nerve VI). Rather, in many cases, the lateral rectus is aberrantly innervated by the oculomotor nerve (cranial nerve III) [8]. Mutations in several genes, including CPA6, have been linked to Duane syndrome [7], [9], [10], [11], [12]. The CPA6 gene is located within a genomic locus named DURS1, previously implicated in Duane syndrome [13], [14]. However, the causative gene at the DURS1 locus has not been definitively identified.

The possibility that a mutant CPA6 might be responsible for axon guidance defects leading to Duane syndrome prompted us to investigate the role of CPA6 in the zebrafish. This model organism enables the easy determination of developmental gene expression patterns and quantitative analysis of eye movement following perturbation of gene expression. Here we describe the cloning of zebrafish CPA6, its enzymatic activity and mRNA distribution during embryonic development. Although the expression pattern of CPA6 is consistent with a role in Duane syndrome, behavioral data show no effect on eye movement upon CPA6 knockdown. We propose that CPA6 may be involved in the etiology of Duane syndrome through expression in a relevant chondrogenic condensation, but dysfunction of additional genes and/or evolutionary modification of axon guidance is required for the manifestation of a phenotype.

## Results

### Identification and spatiotemporal expression of carboxypeptidase genes in the zebrafish

**Figure 1:**
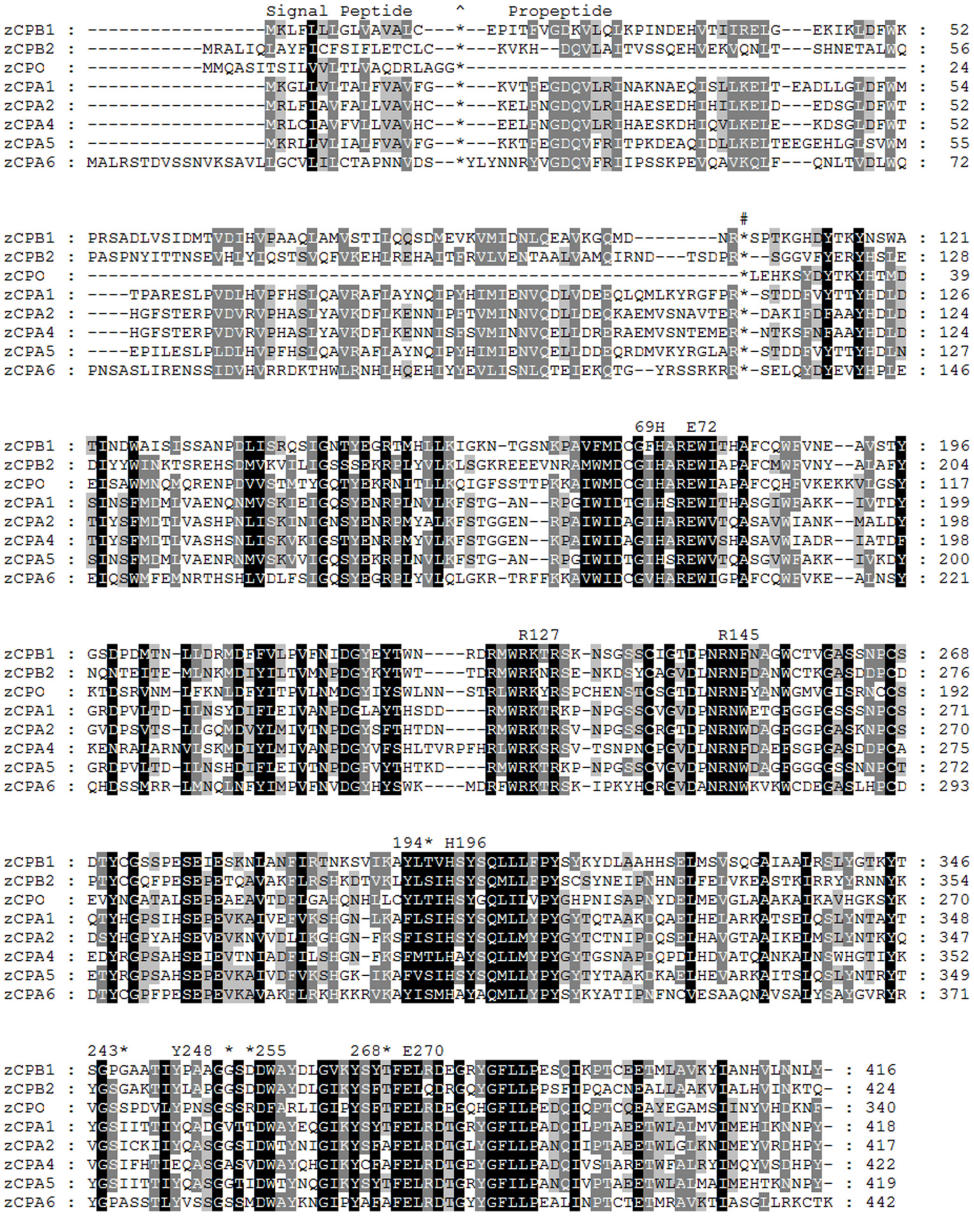
Members of the A/B subfamily of metallocarboxypeptidases identified in the zebrafish. Amino acid sequences of identified CPs were aligned using ClustalW. Greater sequence similarity is indicated by darker background, while locations of signal peptide cleavage, as predicted by SignalP, and of propeptide cleavage, based on experimental evidence for other orthologs, are indicated by a ∧ and #, respectively. Critical active site residues are numbered above the alignment according to their corresponding site in bovine CPA1. Sequences were obtained from the NCBI or Ensembl databases and correspond to the following accession numbers: zCPB1, ENSDARP00000066831; zCPB2, BC095834; zCPO, NM001145629; zCPA1, ENSDARP00000024981; zCPA2, ENSDARP00000064203; zCPA4, ENSDARP00000014821; zCPA5, ENSDARP00000034171; zCPA6, XM001342113.

**Figure 2:**
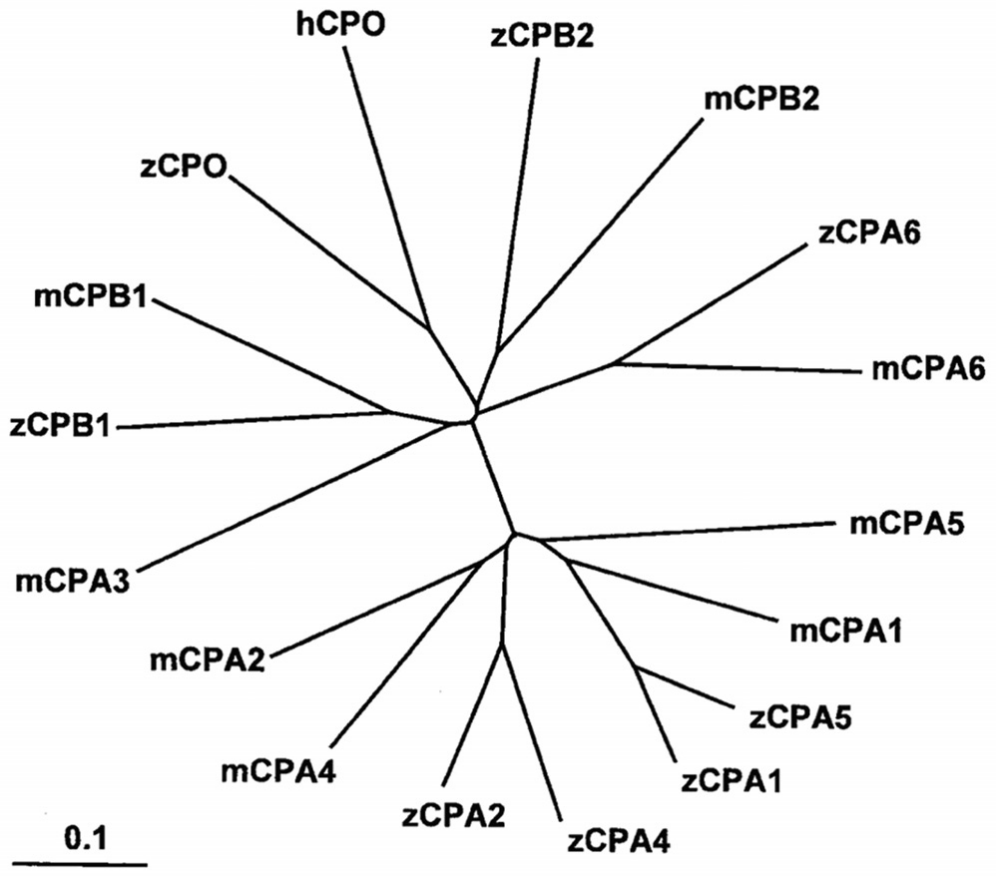
Phylogenetic tree indicating relationships of mammalian and zebrafish A/B-type metallocarboxypeptidases. Sequences were aligned by ClustalW and .dnd files produced to construct a phylogenetic tree. Scale bar indicates substitutions per site.

In order to study the role of CPA6 in zebrafish development, particularly the development of the VIth cranial nerve relevant to Duane syndrome, the zebrafish genome was screened for all A/B-type CPs using a bioinformatics approach. Two CPB-like genes, one CPO-like gene, and five CPA-like genes were found based on similarity to mouse and human sequences (Fig. 1). Most of the zebrafish CPA/B genes were orthologs of mammalian CPA/B genes, based on sequence similarity (Fig. 2). No ortholog of mammalian CPA3 (mast cell carboxypeptidase) was identified by sequence homology. While zebrafish CPA1 and CPA5 amino acid sequences were equally similar to both mouse CPA1 and CPA5, their spatial and temporal expression profiles suggested their respective identities (described below). The same applied to zebrafish CPA2 and CPA4. In addition to overall sequence similarity to mammalian CPA6 orthologs, zebrafish CPA6 contains a methionine (Met387) at a key position in the substrate-binding pocket (equivalent to residue 255 of mature bovine CPA1). This methionine is a defining feature of CPA6 in all known vertebrate orthologs which is not found in other members of the CP family [1].

**Figure 3:**
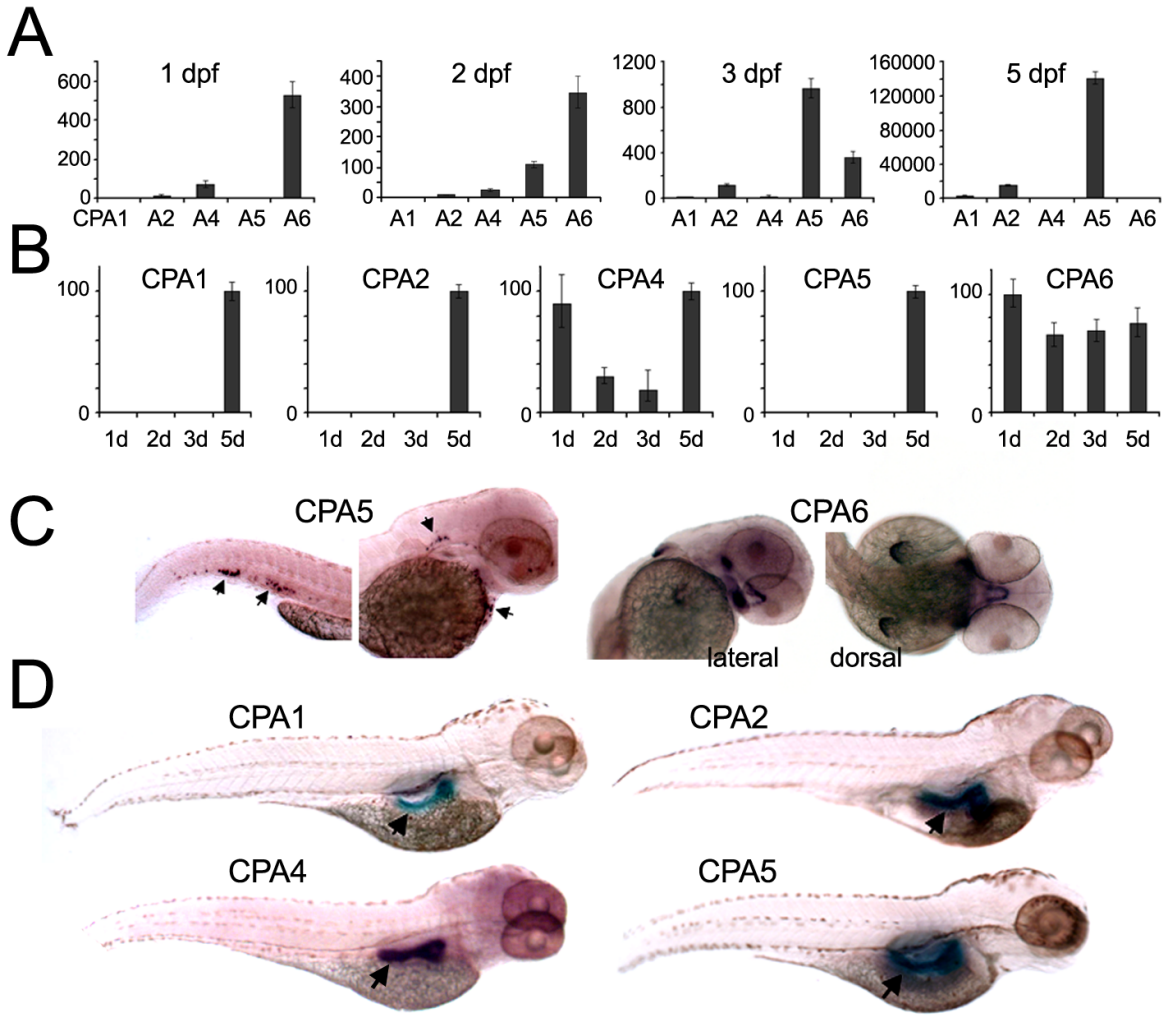
Temporal and spatial expression of zebrafish CPA genes. (A, B) qPCR was performed using cDNA prepared from zebrafish at the indicated developmental time points. All values were normalized to β-actin, and are shown in (A) as expression relative to CPA1 mRNA at 2 dpf, or in (B) as expression of each gene relative to its highest level (100%). (C, D) *In situ* hybridization was used to determine the spatial distribution of CPA mRNA expression. (C) At 2 dpf CPA5 mRNA (arrows) was detected in a mast cell population and CPA6 mRNA was seen in precursor tissues found in the stomodeum, posterior to the eyes, and in the pectoral fin buds. (D) At 4 dpf, mRNA for CPA1, 2, 4, and 5 were expressed in the pancreas.

Temporal and spatial patterns of expression for all zebrafish CPA genes were determined by both quantitative RT-PCR and *in situ* hybridization. Notably, CPA6 was the major CPA gene expressed during embryogenesis at 1 and 2 days post-fertilization (dpf; Fig. 3A). RT-PCR showed CPA6 mRNA to be detectable as early as 11 hpf (data not shown) and maintained at comparable levels from 1 to 5 dpf throughout embryonic development (Fig. 3B). At 2 dpf, CPA6 mRNA was found in the precursors of the stomodeum and pectoral fins and in a condensation posterior to the eyes (Fig. 3C). This distribution, particularly near the eyes and in the pectoral fin buds, is consistent with that seen for CPA6 mRNA in the mouse embryo [1](unpublished data), further confirming a molecular and functional homology. CPA1 and CPA2 mRNAs were both highly expressed in the pancreas of larval zebrafish from 4 dpf (Fig. 3A, B, D), consistent with the pancreatic distribution of the mammalian orthologs. Although RT-PCR showed CPA4 mRNA to be weakly expressed at all time points examined (Fig. 3B), CPA4 mRNA was not detected at any time point by *in situ* hybridization. CPA5 mRNA was localized at 2 dpf to a subpopulation of cells recently identified to be mast cells [15], as well as in the pancreas at 4 dpf (Fig. 3C, D). In fact, both RT-PCR and *in situ* hybridization suggest that CPA5 is the predominant pancreatic CPA in the zebrafish at 3–5 dpf. No other CPA mRNA was found to have an expression profile overlapping with that of CPA6 mRNA.

### Zebrafish CPA6 is an extracellular carboxypeptidase

**Figure 4:**
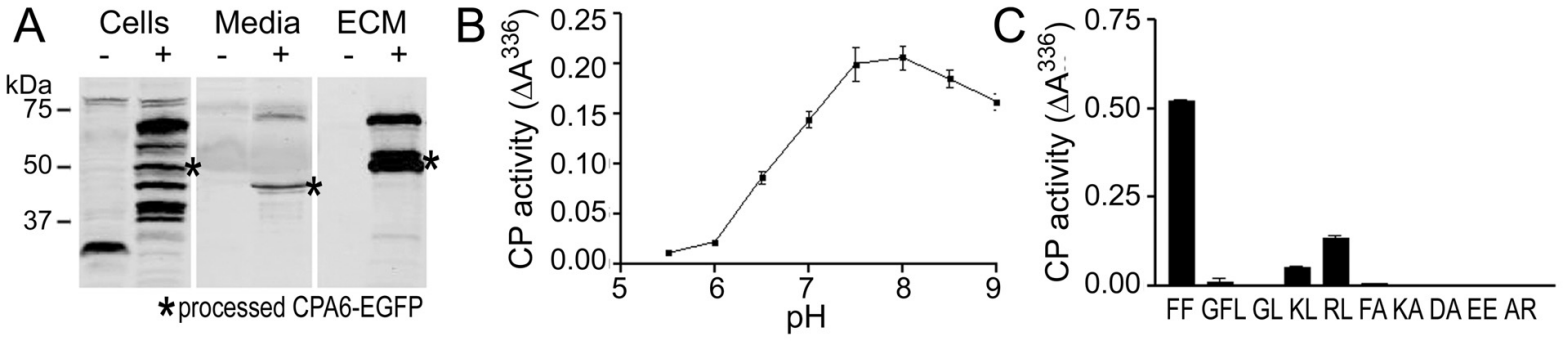
Cellular distribution and enzymatic activity of zebrafish CPA6. (A) EGFP-tagged CPA6 (+) or EGFP only (−) were expressed in HEK293T cells. Media, cell and ECM extracts were resolved by SDS-PAGE and western blotted with an anti-GFP antibody, showing the majority of processed CPA6-EGFP (asterisk) localized to the ECM. (B) ECM from cells transfected with zCPA6 was incubated with fa-FF at the pH values indicated or (C) with a range of commercially available fa substrates at pH 7.5. CP activity was determined by a change in substrate absorbance at 336 nm. Error bars indicate range of duplicate determinations from a representative experiment. Similar results were obtained in two separate experiments.

To determine the enzymatic properties of zebrafish CPA6, the cDNA was cloned into pcDNA3.1. EGFP-tagged and untagged versions were used in subsequent experiments. Upon transfection into HEK293T cells, mature EGFP-tagged CPA6 (65 kDa) was detected by western blot in the ECM, while the proenzyme (78 kDa) was found in cell extracts (Fig. 4A). Unlike that previously reported for human CPA6, a small amount of CPA6 was detected in the media, likely due to the presence of the relatively large C-terminal EGFP tag. The activity of ECM-bound CPA6, both EGFP-tagged (not shown) and untagged, was measured through a standard enzyme assay using fa-FF as a substrate. The pH optimum for zebrafish CPA6 was 7.5–8.0 (Fig. 4B), as previously reported for human CPA6 [6]. Substrates optimal for human CPA6 [6] were also found to be optimal for zebrafish CPA6. Among a series of commercially available substrates, fa-FF and fa–RL were most rapidly cleaved while small amino acids (alanine) and those following a penultimate glycine were not favored (Fig. 4C). Based on the enzymatic properties of CPA6, in addition to its sequence and expression profile, the identified gene is clearly the zebrafish ortholog of CPA6.

### CPA6 is expressed in non-myogenic tissues in the zebrafish

**Figure 5:**
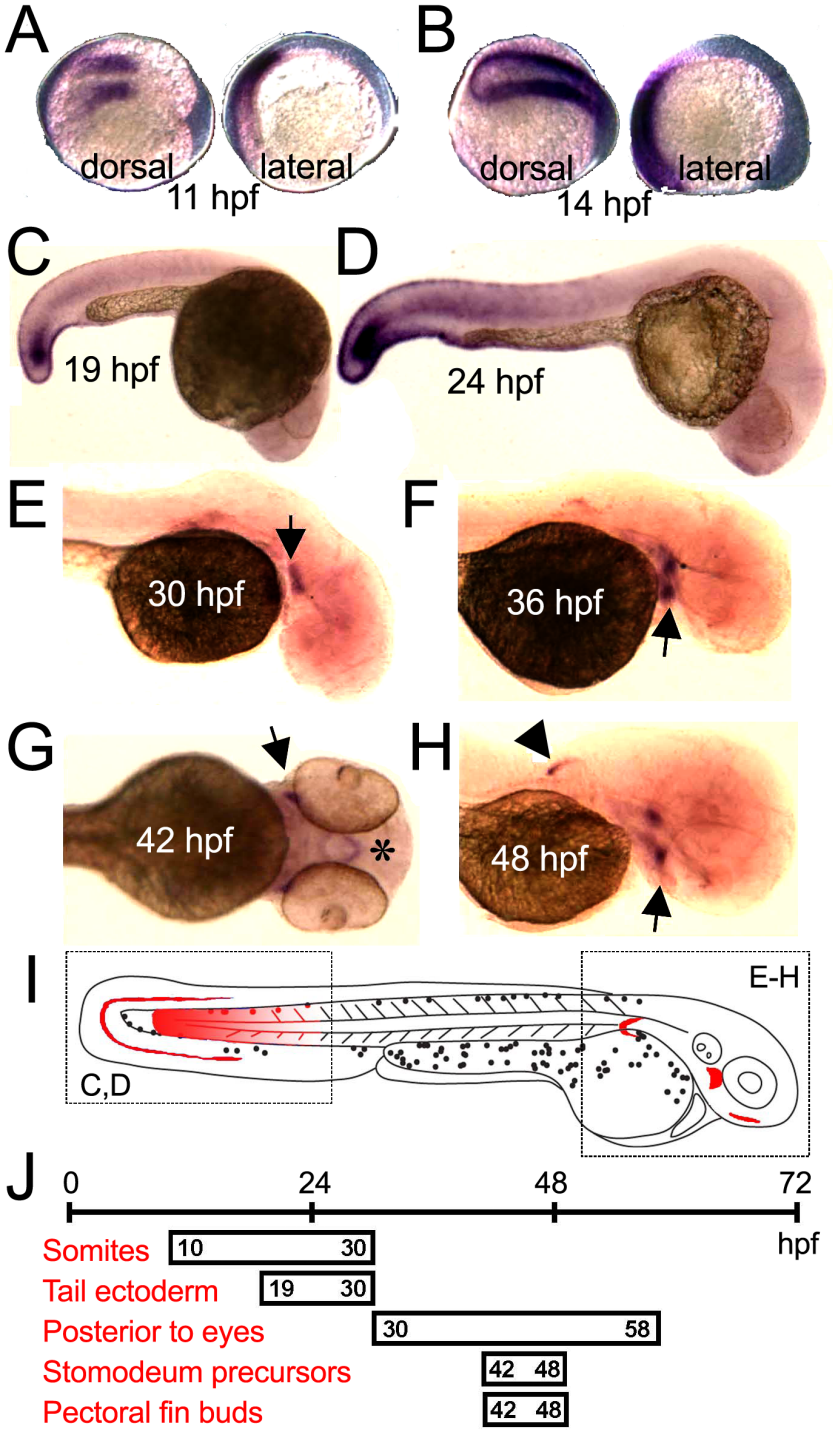
Detailed analysis of CPA6 mRNA expression throughout zebrafish development. *In situ* hybridization indicated CPA6 mRNA (purple) is found in newly formed somites (A–D), ectodermal cells of the tail (C,D), a tissue posterior to the eye (E–H, arrows), the stomodeum (G, asterisk), and the pectoral fins (H, arrowhead). CPA6 expression posterior to both left and right eyes can be seen in F and H. A summary of the spatial (I) and temporal (J) expression of CPA6 throughout zebrafish development is shown.

A detailed *in situ* hybridization time series was performed to determine the spatiotemporal expression profile of CPA6 during zebrafish development. CPA6 mRNA was first seen at 11 hpf in the presomitic mesoderm and the most recently formed somites (Fig. 5A,B). Expression in the somites continued until about 24 hpf (Fig. 5D), with weak staining detected as late as 30 hpf in the tip of the tail. At 19 and 24 hpf CPA6 mRNA was also detected in ectodermal cells of the tail (Fig. 5C,D) and weakly in more anterior somites (Fig. 5D). CPA6 was first localized posterior to the eyes at 30 hpf, with greatest expression seen between 42 and 48 hpf (Fig. 5E–H) which was undetectable by 68 hpf (not shown). During the narrow time window represented by the 42 and 48 hpf time points, CPA6 mRNA was also found in the precursors of the stomodeum (Fig. 5G, asterisk) and the pectoral fins (Fig. 5H, arrowhead). This spatial and temporal pattern of CPA6 gene expression is summarized in Figure 5I–J.

**Figure 6:**
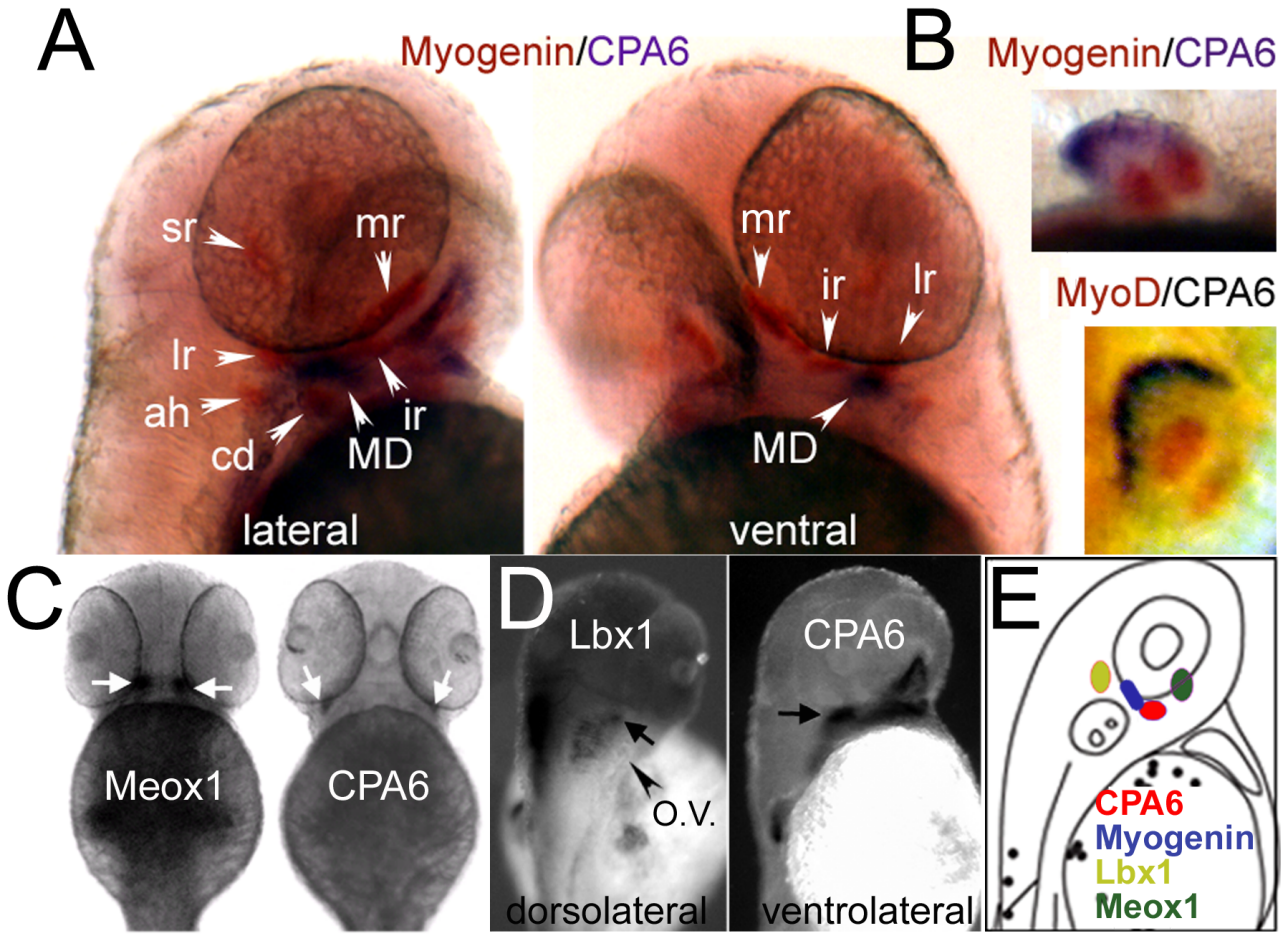
Distribution of CPA6 mRNA compared with tissue-specific markers at 2 dpf. (A) A general marker of muscle precursors, myogenin [16], labels most extraocular muscles (orange) by *in situ* hybridization, but does not co-localize with CPA6 mRNA (purple). (B) Myogenin mRNA, as well as MyoD mRNA, is also found in the pectoral musculature, unlike CPA6 mRNA which is ectodermal. CPA6 mRNA does not co-localize with Meox1 (C), or Lbx1 (D), both putative markers of the lateral rectus muscle in the chick, but likely not in the zebrafish. (E) A schematic of a 2 dpf zebrafish indicates the relative spatial expression of CPA6, myogenin (in lateral rectus), Lbx1 and Meox1. lr, lateral rectus; mr, medial rectus; sr, superior rectus; ah, adductor hyoideus; cd, constrictor dorsalis; MD, myodome precursors; O.V., otic vesicle.

The presence of CPA6 in a condensation posterior to the eye, previously found in the mouse and suggested to be the lateral rectus muscle [1], was of particular interest as CPA6 had been implicated in Duane syndrome [7]. Double *in situ* hybridization of CPA6 with myogenin, a marker of muscle precursors [16], was performed in order to identify this CPA6-expressing tissue (Fig. 6A). When CPA6 expression was compared with the location of extraocular muscle precursors labeled by myogenin [16], it was clear that CPA6 was beside, but not in, lateral rectus myocytes. This suggested the identity of this condensation to be chondrogenic, rather than myogenic, and likely to be the precursor of a structure called the myodome (see discussion). The ectodermal CPA6 expression in the pectoral fin bud, rather than in myogenin- or MyoD-expressing muscle precursors (Fig. 6B), further confirmed the localization of CPA6 to non-myogenic tissues during embryonic development of the zebrafish.

To further confirm the identity of the tissue near the eyes in which CPA6 is found, two putative markers of the chick lateral rectus muscle, Meox1 [17] and Lbx1 [18] were investigated. However, *in situ* hybridization for the zebrafish ortholog of the Meox1 gene showed medial extraocular expression at 2 dpf that clearly was not co-localized with CPA6 mRNA or with myogenin-expressing lateral rectus precursors (Fig. 6C,E). Likewise, at 2 dpf the zebrafish ortholog of Lbx1 was found in the anterior hindbrain as well as in a more lateral group of cells (Fig. 6D, arrow). This domain of expression was clearly more dorsal to that seen for CPA6 and for myogenin-expressing lateral rectus muscle precursors (Fig. 6E). It is likely that these two genes, Meox1 and Lbx1, are not lateral rectus specific markers in the zebrafish.

**Figure 7:**
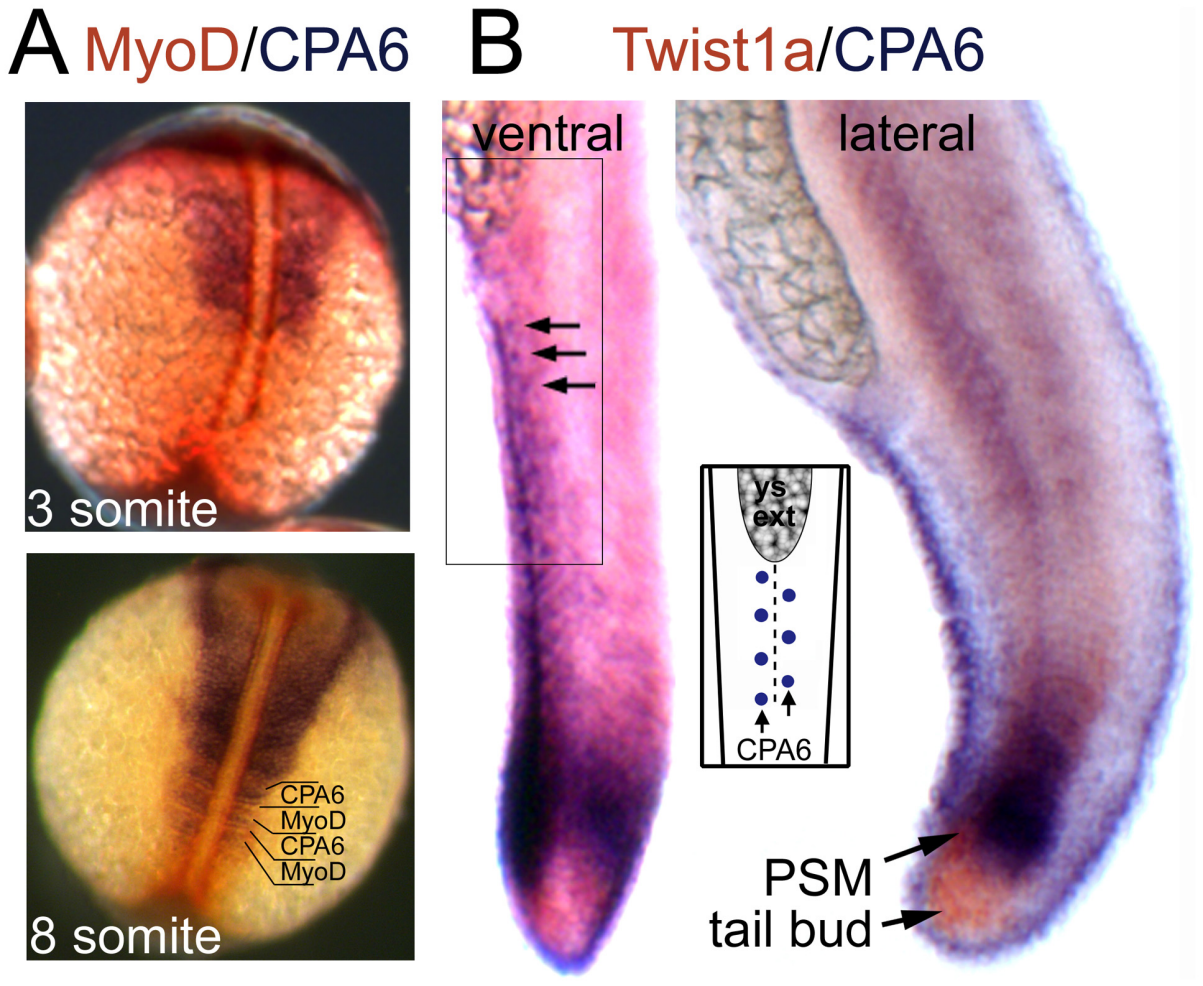
Distribution of CPA6 mRNA compared with somitogenesis markers. *In situ* hybridization was performed with RNA probes specific for (A) CPA6 (purple) and MyoD (orange) at 3 and 8 somite stages (11 and 14 hpf), and (B) CPA6 (purple) and Twist1b (orange) at 24 hpf. Arrows indicate ectodermal cells arranged along the ventral ridge of the tail and expressing CPA6. The regular arrangement of these cells, also found along the dorsal ridge, is illustrated in the inset. PSM, presomitic mesoderm; ys ext, yolk-sac extension.

A closer examination of the distribution of CPA6 in the zebrafish tail was performed through double *in situ* hybridization for CPA6 and MyoD or Twist1b (Fig. 7). MyoD is a transcription factor expressed first in adaxial cells in the medial presomitic mesoderm surrounding the notochord [19]. In a 3-somite embryo (11 hpf, Fig. 7A, top panel), CPA6 mRNA was seen in the lateral somites but not in the medial MyoD-expressing adaxial cells. At the 8-somite stage (14 hpf, Fig. 7A, bottom panel), CPA6 was found in the anterior portion of the most recently formed somites. This pattern was in contrast to MyoD, found only in the fast muscle cells of the posterior half of each somite [20]. Twist1b is a marker of the zebrafish tailbud [21], a region containing progenitor cells which continuously migrate into the presomitic mesoderm for incorporation into somites [22]. Double *in situ* hybridization showed CPA6 to be in the presomitic mesoderm but not in the Twist1b-expressing progenitor cells of the tail bud (Fig. 7B). CPA6 was also clearly localized to a population of regularly arranged ectodermal cells of unknown identity along the ventral and dorsal edges of the tail (Fig. 7B, see inset). The distribution of CPA6 in the tail further illustrates non-myogenic localization and suggests a potential role in regulating migration of somitic precursors.

### Knockdown of zebrafish CPA6 results in normal horizontal eye movements

**Figure 8:**
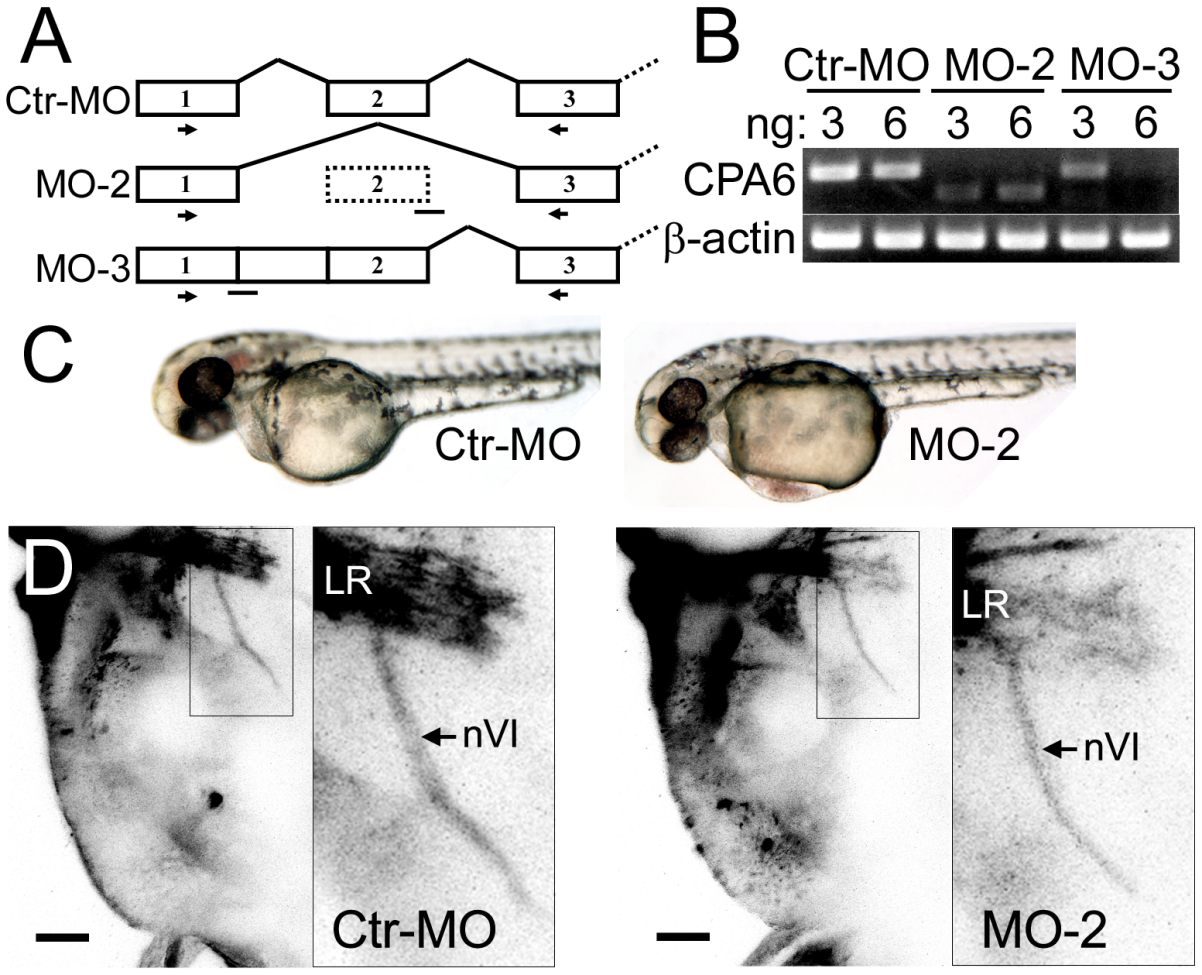
Knockdown of CPA6 gene function. (A) Morpholinos were designed to interfere with normal CPA6 mRNA splicing (Ctr-MO: control morpholino; MO-2 and MO-3: CPA6 specific morpholinos; lines: MO binding sites; arrows: primer binding sites). (B) RT-PCR using RNA extracted from either uninjected or morpholino-injected embryos (3 ng) showed CPA6-specific knockdown. (C) 48 hpf embryos injected with control morpholino and CPA6 MO-2. CPA6 morphants exhibited yolk-sac edema. (D) DiI injected into the lateral rectus muscle (LR) of 8 dpf Ctr-MO and MO-2 CPA6-morphant zebrafish larvae retrogradely labeled the VIth cranial nerve (nVI). Scale bar  = 40 µm.

Two morpholino antisense oligos were designed to mediate knockdown of CPA6. Both were splice-blockers against different exon/intron boundaries (Fig. 8A). RT-PCR using flanking primers revealed near complete knockdown in 2 dpf zebrafish with 3 ng of MO-2 or 6 ng of MO-3 (Fig. 8B). This knockdown was confirmed using quantitative real-time PCR (Fig. S1). Following morpholino injection embryos appeared grossly indistinguishable from controls except for frequent yolk-sac edema beginning at 36 hpf and recovered by 84 hpf (Fig. 8C). As edema is a common side effect of zebrafish injections, we did not pursue this as a reliable phenotype. No defects were seen in other tissues normally expressing CPA6, including the pectoral fin buds and somitic cells, which were analyzed following staining with DAPI (data not shown).

VIth nerve development was assessed following injection of 3 ng control or CPA6-specific MO-2 morpholinos into 1-cell Olig2:EGFP embryos, in which abducens motoneurons are labeled with EGFP from 24 to 58 hpf [23]. As CPA6 was expressed posterior to the eye during this same time period (24–68 hpf, Fig. 5) and morpholinos were found to result in >95% knockdown at 48 hpf (Fig. S1), knockdown of CPA6 was expected to result in a defect in VIth nerve pathfinding if CPA6 was involved. No changes were observed in the outgrowth or trajectory of the abducens nerve (results not shown). Abducens motoneurons are known to complete innervation of the lateral rectus muscle by 72–80 hpf [24], well within the time period of morpholino efficacy. To confirm normal development of the VIth nerve upon CPA6 knockdown as suggested in the Olig2:GFP fish, abducens motor neurons of wild-type fish injected with 3 ng control or CPA6 MO-2 morpholinos were retrogradely labeled at 8 dpf by DiI injection into the lateral rectus muscle (Fig. 8D). This labeling indicated the presence of the VIth nerve at the lateral rectus muscle, the identity of this nerve being confirmed by the observation of retrogradely labeled nuclei in rhombomeres 5 and 6 of the hindbrain.

**Figure 9:**
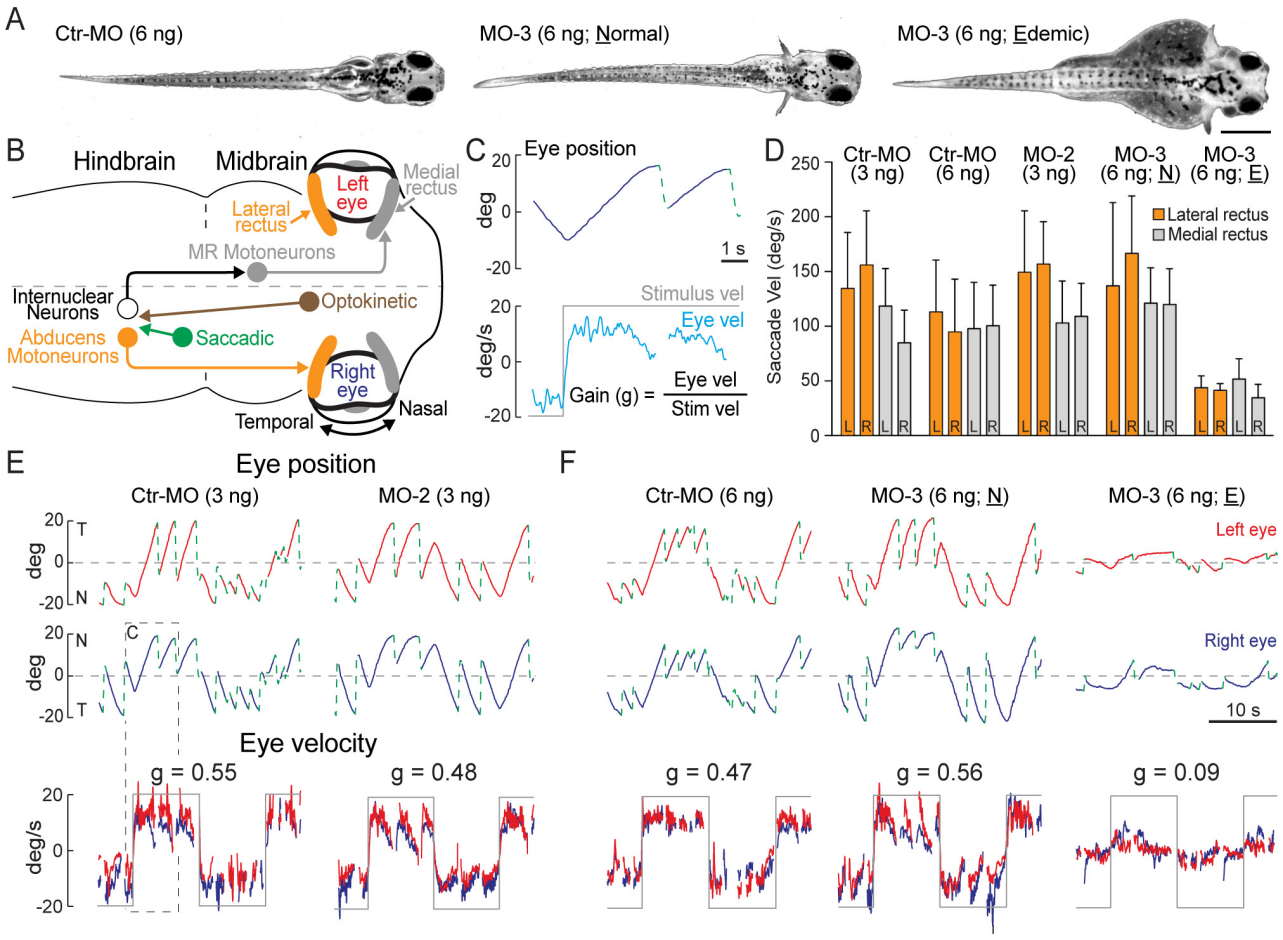
Optokinetic reflex measurement of CPA6 morphants at 5 dpf. (A) Morphology of CPA6 morphants. (B) Schematic illustration of central neural pathways for either lateral or medial rectus mediated optokinetic tracking and saccades. (C) Representative example of eye position (blue trace; top panel), fast phase saccades (green hatching; top panel) and slow phase tracking velocity (cyan trace; bottom panel) in response to ±20°/s velocity steps optokinetic stimuli (grey trace; bottom panel) at 0.0625 Hz. (D) Quantification of saccadic velocity produced by either the lateral (orange bars) or medial rectus muscle (grey bars) in CPA6 morphants and controls. Data are presented as mean ± S.D. (E) Representative eye tracking, saccades and gain in a MO-2 (3 ng) morphant and corresponding control. (F) Optokinetic reflex in MO-3 (6 ng) morphants and corresponding control.

Functional innervation of the lateral rectus muscle was investigated through the quantification of both slow phase optokinetic ‘tracking’ and fast phase ‘saccadic’ eye movement behaviors. In fish, the tracking and saccadic pathways are separate but converge on the abducens internuclear neurons and abducens motoneurons in the hindbrain as illustrated in Fig. 9B. Abducens internuclear neurons project to the contralateral medial rectus motoneurons/muscle while the abducens motoneurons project to the ipsilateral lateral rectus muscle directly (Fig. 9B). Because the majority of the CPA6 morphants were morphologically indistinct from uninjected controls, optokinetic reflex (OKR) measurements provided a direct assessment of the general integrity of the responsible central pathways as well as the motoneuron innervation of the eye muscle.

OKR was measured in larval zebrafish. As MO-2 (3 ng) and MO-3 (6 ng) morphants showed maximal CPA6 knockdown (see Fig. 8B) these were chosen for eye movement analysis at 5 dpf, at which time lateral rectus muscle innervation was complete and the OKR was robust [25] (Fig. 9). From 4 dpf onwards, all Ctr-MO (3 or 6 ng), MO-2 (3 ng) and MO-3 (3 ng) injected larvae were grossly indistinguishable from uninjected controls (Fig. 9A) and responded to tactile, auditory and visual stimuli with avoidance behavior (data not shown). Injection of CPA6 morpholino at a higher dose (6 ng) resulted in a significant number of morphants continuing to be edemic at 5 dpf (MO-2, ∼80%; MO-3, ∼30%, Fig. 9A).

In response to drum velocity steps of ±20°/s, all 5 dpf Ctr-MO and CPA6 morphants exhibited visual tracking of the drum and resetting saccades (Fig. 9C–F). Spontaneous saccades were also observed in the dark (data not shown). Eye velocity was quantified in 947 saccades from 16 animals in 5 treatment paradigms to investigate the potential effects of CPA6 knockdown. Saccadic velocity was similar in Ctr-MO (3 or 6 ng), MO-2 (3 ng) and MO-3 (6 ng; Normal) injected fish. In general, edemic MO-3 (6 ng) morphants exhibited significantly lower saccadic velocity (p<0.001) as determined by a two-way ANOVA analysis with Bonferroni posttests. Within each group of morphants, saccadic velocity was comparable when produced by either the medial or lateral rectus muscle (Fig. 9D).

A measurement of OKR tracking performance is the gain (g), which is the ratio of eye velocity to stimulus velocity [25]. In all morphants (except 6 ng MO-3, Edemic), the gain ranged from 0.37 to 0.56 (Fig. 9E–F). These values are comparable to previously reported measurements [25]. In edemic MO-3 (6 ng) morphants, OKR performance was lower than in control cases as reflected in the reduced saccadic velocity (Fig. 9D) and gain (Fig. 9F); however, both slow and fast phases were still observed. In all cases, OKR of both left and right eyes and performance of both medial and lateral rectus muscles were similar within a group of CPA6 morphants (Fig. 9). Eye movement analysis was repeated at 8 dpf and the data were statistically similar to the results at 5 dpf (data not shown). In summary, CPA6 knockdown in zebrafish did not result in any lateral rectus specific defects in horizontal eye movement.

## Discussion

CPA6 is a well-conserved enzyme among vertebrates in terms of molecular properties and expression pattern. It is not duplicated in the zebrafish genome, although the current zebrafish genome assembly, Zv8, suggests that CPA6 genes exist on both chromosomes 8 and 24. This annotation is likely caused by a genome assembly error, as analysis of these two putative genes indicates 100% identity through all coding and non-coding sequence, including all introns. Zebrafish CPA6 is secreted and binds tightly to the extracellular matrix, where it is able to cleave C-terminal hydrophobic amino acids (Fig. 4). These properties are very similar to those of the human ortholog [6]. The distribution of CPA6 mRNA in embryonic zebrafish tissues (Fig. 5) is also largely conserved when compared with that in the mouse [1]. CPA6 is expressed in both the zebrafish and mouse apical ectodermal ridge of the pectoral fin/limb buds and posterior to the eye. Expression of CPA6 during mouse somitogenesis has not been investigated.

The expression pattern seen for zebrafish CPA6 is not shared by any of the other four CPA-like enzymes found in the zebrafish (Fig. 3). Both CPA1 and CPA2 are detected in the pancreas after 3 dpf, consistent with their known roles as pancreatic digestive enzymes [26]. Although no CPA3 (mast cell carboxypeptidase) was identified by sequence homology in the zebrafish, CPA5 appears to take on this role, with expression found in a population of cells recently identified as mast cells [15]. The weak expression of CPA2 and CPA4 detected by RT-PCR early in development, and of CPA4 and CPA5 detected by *in situ* hybridization in the pancreas later in development, is consistent with previous reports of weak embryonic expression (of unknown function) of human orthologs [27], [28]. For the most part, each CPA gene exhibits distributions consistent with its mammalian ortholog, yet distinct from that of CPA6.

Of particular interest to this study was the conserved expression of CPA6 posterior to the eye, consistent with a role for CPA6 in the etiology of Duane syndrome. Although previous observations suggested that CPA6 was expressed in lateral rectus muscle precursor cells [1], it is difficult to distinguish these cells from developing chondrogenic tissue without markers. Based on the careful analysis of CPA6 distribution in zebrafish, it does not appear that CPA6 is expressed in lateral rectus muscle precursor cells or in any other myogenic cell population (Figs. 6 and 7). Rather, the condensation near the eye in which zebrafish CPA6 is found is consistent with developing chondrogenic tissue. CPA6 is found in bony/chondrogenic tissues (basioccipital cartilage, vertebrae, and ribs) in the mouse also [1]. In teleost fish, the chondrogenic tissue found next to the lateral rectus muscle subsequently forms the walls of a compartment known as the myodome.

The myodome is a hollow tube formed at the base of the teleost cranium inside the parasphenoid and basioccipital bones caudal to the eye [29]. Because the lateral rectus muscle is encompassed by the myodome, the VIth nerve must pass through this structure in order to innervate its target [30], [31]. The myodome appears to be unique to fishes, but with a possible functional equivalent in the cavernous and intercavernous sinuses of man [31]. The bones making up the walls of the myodome have a novel paraxial mesoderm embryonic origin in the chick, unlike most other components of the cranium which originate from migratory neural crest cells [32]. The expression of CPA6 in this tissue suggests a role for CPA6 in the unique specification of cartilaginous tissue from paraxial mesoderm which may be linked to a phylogenetic remodeling of this tissue to enable the VIth nerve to pass and successfully find its target muscle (see below). However, morpholino-mediated knockdown of CPA6 did not result in any visible defects in the trajectory of the VIth nerve nor in any behavioral eye movement defects specific to either the medial or lateral rectus muscle.

The conserved expression of CPA6 in cartilaginous precursors posterior to the eye, however, strongly suggests a role in the development and innervation of the lateral rectus and in the etiology of Duane syndrome. This conundrum between the specific localization of CPA6 adjacent to the lateral rectus muscle and yet lack of a behavioral phenotype upon knockdown might be explained in several ways. First, new mechanisms for VIth nerve development may have evolved in mammalian systems that involve the phylogenetic remodeling of cartilaginous/skeletal elements through the actions of CPA6. Alternatively, CPA6 may have taken on a role in mammalian axon pathfinding that simply is not present in teleost fish. Another possibility is that developmental and molecular characteristics of the abducens motor neurons and the VIth nerve itself may have evolved. This is suggested by anatomical differences between teleost and mammalian abducens nuclei. Abducens motoneurons arise from two nuclei found in hindbrain rhombomeres (r)5 and r6 in the zebrafish, while mammalian abducens motor neurons arise from only one nucleus found in r5 [33]. Axons of r5 abducens motoneurons, the sole population in mammals, may require CPA6 for guidance while axons of r6 motoneurons, when present in teleost fish, may be able to reach their target in a CPA6-independent manner and provide guidance for other CPA6-dependent axons. However, no anatomical or physiological differences between r5 and r6 abducens motor neurons have yet to be discovered [34].

Another possibility for the lack of a CPA6 knockdown phenotype may be the involvement of other genes, acting in a compensatory manner in the zebrafish, or defective in reported cases of Duane syndrome but yet undetected. Recent reports have suggested that CPA6 may not be the sole causative Duane syndrome gene on chromosome 8, but the duplication of another more centromeric gene, CHD7, may be involved [35], [36]. The reported chromosomal translocation disrupting the 5′ end of the CPA6 gene in a Duane syndrome patient [7] might also affect promoter/enhancer elements of neighboring genes. The CPA6 gene is arranged in a head-to-head fashion with the DEPDC2 gene, which encodes a Rac-GEF with broad distribution throughout the zebrafish head (unpublished results). This gene is of note due to the recent identification of activating mutations in the alpha2-chimaerin gene in patients with familial Duane syndrome [10], [37]. Activating mutations in alpha2-chimaerin, a Rac-GAP, might correspond to decreased expression of DEPDC2, a Rac-GEF. Decreased expression of both CPA6 and DEPDC2 might produce a cumulative effect resulting in a Duane syndrome-like phenotype. While this appears to be an excellent hypothesis, knockdown of the DEPDC2 gene along with CPA6 in the zebrafish, using specific splice-blocking morpholinos, did not result in any noticeable defects in eye abduction (data not shown).

In conclusion, a role for CPA6 in the etiology of Duane syndrome is here supported by its expression in a functionally unique and relevant chondrogenic precursor. Although knockdown of CPA6 in zebrafish did not lead to a phenotype specific to lateral rectus innervation, the conservation of molecular function and expression of this enzyme across divergent vertebrate species suggests an important function. Indeed, if CPA6 is found to be involved in the etiology of Duane syndrome, our data suggest that this syndrome is not always myogenic or neurogenic, but can also be a chondrogenic disorder.

## Materials and Methods

### Ethics Statement

All experiments were performed in strict accordance to standard guidelines for zebrafish work and approved by the Animal Institute Committee at Albert Einstein College of Medicine (protocol #20080311).

### Zebrafish care

Zebrafish were maintained under standard conditions as described previously [38]. Zebrafish embryos were injected with morpholino oligonucleotides between 1 and 8-cell stages and were maintained at 28.5°C in egg water [39]. Morpholinos were obtained from Gene Tools, LLC, and dissolved in distilled water. Ctl-MO: GeneTools standard control oligo; MO-2: AGAAAATAAGGGACTCACTGTCAAG; MO-3: GAATGAATGAAACACTTACCCGACG.

### Cell Culture and Transfection

HEK293T cells (ATCC) were cultured in Dulbecco's Modified Eagle's Medium supplemented with 10% fetal bovine serum and penicillin/streptomycin at 37°C and 5% CO_2_. Cells were transfected with HEKFectin (BioRad) according to the manufacturer's protocol.

### Cell lysis and Western Blotting

Medium was removed from plates and cells washed gently with phosphate buffered saline (PBS). Cells were scraped from the plate and lysed in 1% SDS. Following removal of all cells, the plate was washed several times with PBS and extracellular matrix (ECM) proteins were extracted with hot SDS-PAGE sample buffer added directly to the plate. Equal volumes of each extract and medium were run on a 10% SDS polyacrylamide gel (Biorad), proteins were transferred to nitrocellulose and Western blotting performed with rabbit anti-GFP primary antibody (1∶1000) and IRdye800-congugated anti-rabbit secondary antibodies (Rockland, 1∶3000). Images were obtained on a LiCor Odyssey imaging system.

### Carboxypeptidase Assays

3-(2-furyl)acryloyl-peptide (fa) substrates (Bachem) were dissolved in 50 mM Tris-HCl pH 7.5 (at 37°C) and 150 mM NaCl to a concentration of 0.5 mM. For pH optimum determination, substrate was dissolved in 50 mM Tris-acetate buffer containing 150 mM NaCl at the indicated pH values. HEK293T cells were plated in 6-well plates and 24 hours later transfected with CPA6 expression plasmid. Cells were removed 24–48 hours after transfection and wells washed 3 times with 25 mM Tris pH 7.5 containing 150 mM NaCl. One milliliter CP substrate was added and plates were incubated with shaking at 37°C in a humid chamber for 1–6 hours. All assays compared wells in which cells were transfected with CPA6 expression plasmid with untransfected wells. Cleavage of substrate was measured by a decrease in absorbance at 336 nm.

### RT-PCR and cloning of cDNAs

RNA was extracted from zebrafish embryos using the RNeasy Mini kit (Qiagen) according to the protocol for animal tissues. RNA quality was assessed by formaldehyde gel electrophoresis. First-strand cDNA synthesis was performed using the Superscript III First Strand Synthesis System (Invitrogen), followed by amplification of desired cDNA by PCR with Platinum® Pfx DNA polymerase (Invitrogen) using a program of 94° for 3.5 min, followed by 35 cycles of 94° for 15 s, 55° for 30 s, and 68° for 2 min. Quantitative real-time PCR was performed on an ABI 7900 using Power SYBR® Green PCR master mix (Applied Biosystems). Data were normalized to β-actin expression and shown as relative expression using the 2^−ΔΔCT^ method. For cloning purposes, primers (sequences available upon request) contained restriction sites. Amplicons obtained from PCR reactions were digested with appropriate restriction enzymes and ligated into a plasmid, followed by verification by sequencing. RT-PCR for expression analysis was performed using the Superscript III One-Step RT-PCR kit from Invitrogen.

### *In Situ* Hybridization

Probes for *in situ* hybridization were generated by *in vitro* transcription from a linearized plasmid template using a Fluorescein or DIG RNA Labeling Kit (Roche) with T3 or T7 RNA polymerase, according to the manufacturer's protocol. Lithium chloride-precipitated RNA probe was redissolved in water and quality assessed by formaldehyde gel electrophoresis. Zebrafish embryos were fixed in 4% paraformaldehyde in PBS overnight at 4°C and processed for *in situ* hybridization according to standard protocols [40], using a probe hybridization temperature of 70°C. Embryos were finally incubated with alkaline phosphatase substrates BM purple (Roche) or INT/BCIP (Roche) in coloration buffer for 20 minutes to 10 hours at room temperature, reactions stopped by incubation in 0.1M glycine pH 2.2 for 10 minutes and embryos transferred to 70% glycerol for imaging.

### Retrograde Labeling

5–8 dpf zebrafish were mounted with ventral side up in 1% low-melting point agarose. DiI (Invitrogen), dissolved in methanol, was pressure injected into the lateral rectus muscle. 24 hours later images of the retrogradely labeled abducens nerve were collected on a LSM 510 confocal microscope.

### Horizontal optokinetic reflex (OKR) assessment

At 5 and 8 dpf, larvae were mounted in low-melting agar and OKR was induced by visual stimuli of ±20°/s velocity steps using a drum with a stripe frequency of 15.5°. [25], [41]. Measurement and data processing was performed using custom routines in MATLAB (MathWorks, Natick, MA; courtesy of Dr. James Beck) and Prism (GraphPad Software Inc., La Jolla, CA).

## Supporting Information

Figure S1 Knockdown of CPA6 does not significantly affect expression of other zebrafish CPA genes. Six nanograms of indicated morpholinos were injected and RNA extracted at 2 dpf. Real-time PCR indicated near complete knockdown of CPA6 by both morpholinos. CPA4 and CPA5 mRNAs appeared slightly affected by MO2 morpholino but not by MO3 morpholino. Expression of CPA1 and CPA2 mRNAs were not affected by either morpholino.
(0.04 MB TIF)
