# Supplementary figures and images for: Carboxypeptidase A6 in Zebrafish Development and Implications for VIth Cranial Nerve Pathfinding
Source: PLoS One. 2010 Sep 24;5(9):e12967. doi: 10.1371/journal.pone.0012967 (PMC2945764; doi:10.1371/journal.pone.0012967)

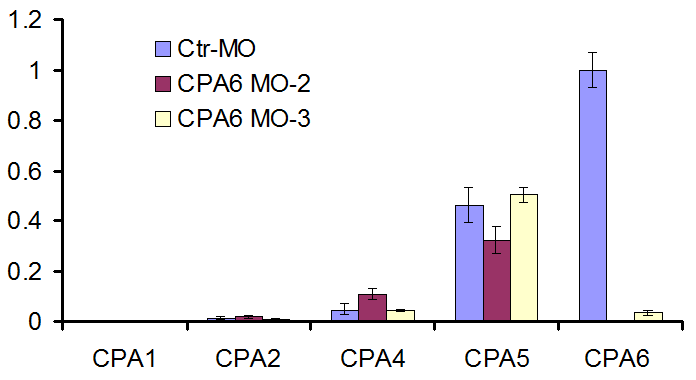

Supplement: Figure S1 — Knockdown of CPA6 does not significantly affect expression of other zebrafish CPA genes. Six nanograms of indicated morpholinos were injected and RNA extracted at 2 dpf. Real-time PCR indicated near complete knockdown of CPA6 by both morpholinos. CPA4 and CPA5 mRNAs appeared slightly affected by MO2 morpholino but not by MO3 morpholino. Expression of CPA1 and CPA2 mRNAs were not affected by either morpholino. (0.04 MB TIF) [file pone.0012967.s001.tif]
